# Assessing the effects of prosthetic foot stiffness and foot preference on stability, balance confidence, and satisfaction in transtibial prosthesis users: Protocol for a randomized, participant-masked crossover trial using a ‘test-drive’ strategy

**DOI:** 10.1371/journal.pone.0334497

**Published:** 2025-10-22

**Authors:** Tyler K. Ho, Elizabeth G. Halsne, Sara R. Koehler-McNicholas, Andrew H. Hansen, Andrew Sawers, Joshua M. Caputo, Carl S. Curran, Alexandria Lloyd, Juan Cave, David C. Morgenroth

**Affiliations:** 1 Center for Limb Loss and MoBility, VA Puget Sound Health Care System, Seattle, Washington, United States of America; 2 Department of Rehabilitation Medicine, University of Washington, Seattle, Washington, United States of America; 3 Rehabilitation & Engineering Center for Optimizing Veteran Engagement & Reintegration (RECOVER), Minneapolis VA Health Care System, Minneapolis, Minnesota, United States of America; 4 Department of Family Medicine and Community Health, Rehabilitation Science Program, University of Minnesota, Minneapolis, Minnesota, United States of America; 5 Department of Kinesiology, University of Illinois Chicago, Chicago, Illinois, United States of America; 6 Human Motion Technologies LLC d/b/a Humotech, Pittsburgh, Pennsylvania, United States of America; Public Library of Science, UNITED KINGDOM OF GREAT BRITAIN AND NORTHERN IRELAND

## Abstract

**Introduction:**

With an appropriate prescription, the use of a lower limb prosthesis can help mitigate mobility limitations and increased risk of falling for people with lower limb amputation. Prosthetic feet cannot replicate all the functions of a biological foot-ankle. Different feet have different designs and properties, and therefore there are functional trade-offs. There is insufficient evidence as to the effects these different prosthetic foot properties have on users’ stability and balance, which would be helpful to guide clinical prosthesis prescription. Prosthesis users also rarely have opportunities to try walking with different prosthetic feet to give experiential input during the prescription process. Therefore, this study aims to determine 1) the effects of prosthetic foot stiffness on stability in lower limb prosthesis users while walking on varying terrains, and 2) whether a brief ‘test-drive’ strategy for selecting prosthetic feet can be used to predict longer term stability, balance confidence, and foot preference outcomes in lower limb prosthesis users.

**Methods and Materials:**

In this multisite, participant-masked, randomized cross-over study, participants with unilateral, transtibial amputation will walk on different treadmill conditions (flat, incline, cross-slopes, uneven ground) with a variety of commercially-available prosthetic feet (‘*actual*’) and corresponding ‘*emulated*’ prosthetic feet in the laboratory. Participants will also wear the *actual* prosthetic feet at home and in the community for one week at a time. After each community trial, participants will return to the laboratory to complete walking trials on different terrains and at a range of speeds while we collect kinematic data. We will assess the effect of prosthetic foot stiffness on biomechanical and self-reported measures of stability. We will also assess how well brief ‘test-drive’ trials of walking with different prosthetic feet can predict longer-term self-reported measures of stability, balance confidence and preference.

**Trial registration:**

This study was prospectively registered at www.clinicaltrials.gov (Clinical Trials Study ID: NCT05473065). Study start date: March 1, 2024.

## Introduction

Lower limb amputation results in a range of mobility limitations [1–3] and more than 50% of lower limb prosthesis (LLP) users experience at least one fall per year [4–8]. The incidence of falls-related injuries among LLP users is more than 45 per 100,000 person-days [8], which is greater than the incidence of major falls-related injuries among hospitalized people aged 75 and older (roughly 30 injuries per 100,000 person-days) [9]. With an appropriate prescription, the use of a LLP can help restore mobility and mitigate some of the fall risk for people with an amputation [10,11]. The prosthetic foot is a vital component of a LLP. There are hundreds of different commercially available prosthetic feet, although none can effectively replicate the diverse functionality of the biological foot and ankle [12–15]. The materials, geometry, and other design features of prosthetic feet determine which functional uses they might optimize, and what functional trade-offs exist (e.g., more stable feet may have less energy return) [16].

Given that the needs, goals, and priorities of prosthesis users vary considerably across individuals [17], it is important to match each LLP user to a prosthetic foot design that can maximize the user’s unique abilities and functional goals. To choose a prosthetic foot as part of a user’s LLP prescription, clinicians often rely on their familiarity with specific feet, manufacturer claims about foot performance, and their knowledge of biomechanical principles [18]. However, there is limited evidence as to the effect of different mechanical properties of prosthetic feet on LLP users’ stability and balance while walking in a range of environments. Prior work has shown that prosthetic feet with lower rotational stiffness and greater motion promote earlier flat foot during the initial stance phase and have been associated with improved perceived stability [19,20]. Based on these results, we expect that sagittal and coronal plane prosthetic foot rotational stiffness are likely to be inversely associated with relative stability while walking on inclines, cross-slopes and other uneven walking surfaces. Therefore, the first aim of this study is 1) to determine the effects of sagittal and coronal plane prosthetic foot stiffness on stability measures in LLP users walking on varying terrains.

In addition to gaining a better understanding of the relationship between prosthetic foot stiffness and functional and stability outcomes, it would also be helpful to provide LLP users the opportunity to provide feedback about different prosthetic feet while walking over various terrains. LLP users rarely have such opportunities to try walking with different types of prosthetic feet in the clinic [17,21]. Yet, both patients and clinicians acknowledge the importance of the user’s input and experience to a successful LLP prescription [22–24]. In order to determine the predictive validity of a ‘test-drive’ strategy for prosthetic foot selection, the second aim of this study is 2) to determine whether a brief trial of prosthetic feet in the laboratory can be used to predict longer term stability, balance confidence, and foot preference outcomes of LLP users.

## Methods and analysis

### Study design

**Fig 1:**
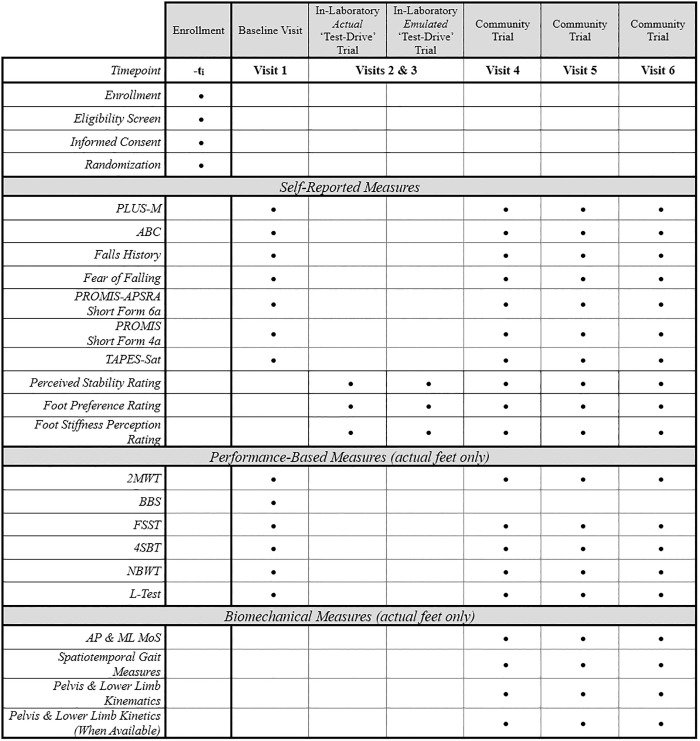
Participant timeline. This table describes the outcomes that will be collected at each study visit.

**Fig 2:**
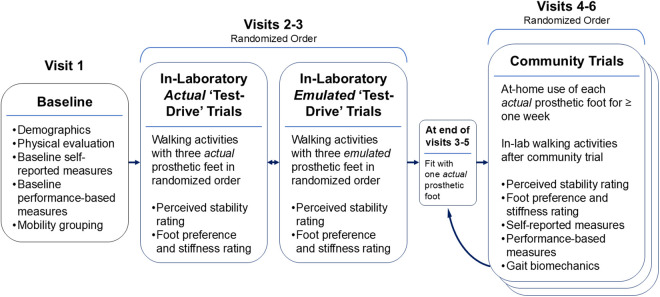
Study schematic. Overview of study visits and the activities that will be performed during each.

This is a multisite, participant-masked, randomized cross-over study. Outcome measures will include self-reported surveys, performance-based tests, and biomechanical gait variables. Participants will walk on different treadmill conditions (flat, incline, cross-slopes, uneven ground) with a variety of commercially-available prosthetic feet (‘*actual*’) and corresponding ‘*emulated*’ prosthetic feet (using a robotic prosthetic foot emulator, see details below) in the laboratory. Participants will also wear the *actual* prosthetic feet at home and in the community for one week at a time (Figs 1–2).

#### Recruitment.

We will recruit participants who have a unilateral, transtibial amputation and have been using a LLP for at least six months. We will conduct data collections at the Veterans Affairs Puget Sound Health Care System in Seattle, WA and at the Minneapolis Veterans Affairs Health Care System in Minneapolis, MN. For participant recruitment, we will identify LLP users using electronic medical records or participant registries and will contact candidate participants to discuss their interest in participation. Additionally, we will provide recruitment materials (i.e., flyers) to local clinics to assist in recruitment efforts. LLP users who respond to these recruitment materials will be provided with information on the study and will be screened for participation eligibility.

We began initial recruitment and data collection on March 1, 2024. We estimate that we will complete participant recruitment by June 1, 2026 and complete data collection by July 31, 2026. Results will be disseminated after all data have been analyzed.

#### Inclusion/Exclusion criteria.

We will include participants that 1) are 18–89 years old, 2) have a unilateral, transtibial amputation, 3) are currently using a prosthesis and have used one for at least six months, 4) have a comfortably fitting prosthetic socket, 5) can walk continuously for at least two minutes at a time, and 6) are able to read and communicate in English. We will exclude participants that 1) are pregnant, 2) have an arm amputation or major amputation on the contralateral leg, and 3) weigh more than 263lbs (113 kg), due to the manufacturer weight limit of the study equipment (Humotech Caplex™ System).

### Intervention

#### Prosthetic feet.

We selected five prosthetic foot models to represent a clinically appropriate and commonly prescribed group of commercially-available (‘*actual*’) prosthetic feet with a range of sagittal and coronal plane stiffness properties. These foot models are from a variety of manufacturers, have different material properties (e.g., fiberglass versus carbon fiber), geometries (e.g., keel shape), and elastic components (e.g., fiber straps versus elastomeric bumpers). Consequently, these feet exhibit a range of behaviors in the sagittal and coronal planes. These feet will be used with each participant’s prescribed socket and interface/suspension.

In order to enhance the applicability of our findings, we will include participants with a range of mobility levels, and sort them into either a *low mobility* or a *high mobility* group (details below). *Low mobility* participants will walk on the following three prosthetic feet: A) a non-energy storage and return (non-ESR) flexible keel foot, B) a non-ESR multiaxial foot, and C) a split toe/heel ESR foot. *High mobility* participants will also walk on C) a split toe/heel ESR foot, as well as the following two prosthetic feet: D) an ESR multiaxial foot, and E) an extended keel ESR foot.

#### Multiaxial prosthetic foot emulator.

**Fig 3:**
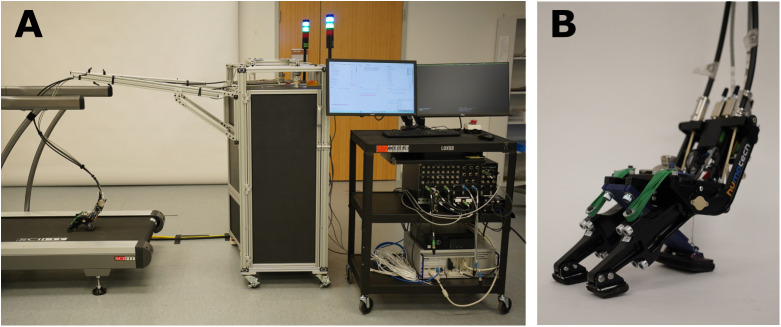
Prosthetic foot emulator. (A) Two off-board actuators control the sagittal and coronal stiffness properties of the (B) prosthetic end effector by manipulating the torque produced by the two toes of the end effector.

We will use a two degree-of-freedom prosthetic foot emulator (PFE)—the Humotech Caplex™ System (Humotech, Pittsburgh, PA) [25] configured with a multiaxial prosthetic foot end effector (PRO-003) which enables motion in the sagittal and coronal planes (Fig 3). The PFE has two off-board actuators (Fig 3A) that control the rotational stiffness of two ‘toes’ on the prosthetic foot end effector (Fig 3B). The PFE is adjusted to match the size, heel stiffness and weight of each *actual* prosthetic foot. The PFE end effector will be attached to each participant’s prescribed socket and interface/suspension and be aligned in the same manner as the *actual* feet (details below). In addition to walking in the laboratory with the *actual* feet detailed above, participants will walk with the PFE while it emulates the sagittal and coronal properties of each of the three feet (one at a time) assigned to their mobility group.

### Human subjects protocol

This study consists of six separate visits (Fig 2) over the course of approximately 1–2 months. During these visits, participants will complete in-laboratory ‘test-drive’ trials (details below) with *actual* and *emulated* versions of three prosthetic feet in a randomized order. Participants will also complete one-week community trials with one *actual* foot at a time in a randomized order (one week with each of three different prosthetic feet).

#### Baseline visit.

During visit 1, participants will provide written informed consent prior to any study-related activities. A certified prosthetist will then evaluate each participant, and we will collect pertinent amputation-related history and demographics information. Then we will collect self-reported measures (detailed below) and performance-based measures (detailed below), and determine participants’ comfortable, slow, and fast walking speeds while walking on a treadmill with their prescribed prosthetic foot. They will also be given the opportunity to walk with the PFE to become acclimated during visit 1.

#### Mobility group assignment.

During the first visit, participants will complete several performance-based measures (details below), which includes the two-minute walk test (2MWT) [26]. Participants that walk at least 375 feet (113m) will be categorized as *high mobility,* while those that walk less than 375 feet (113m) will be categorized as *low mobility.* Previous work has shown that a cutoff of 113m distinguishes between limited community ambulators (i.e., *low mobility*) and fully functional community ambulators (i.e., *high mobility*) within prosthesis users [27].

#### In-laboratory *actual* prosthetic foot ‘test-drive’ trials.

**Fig 4:**
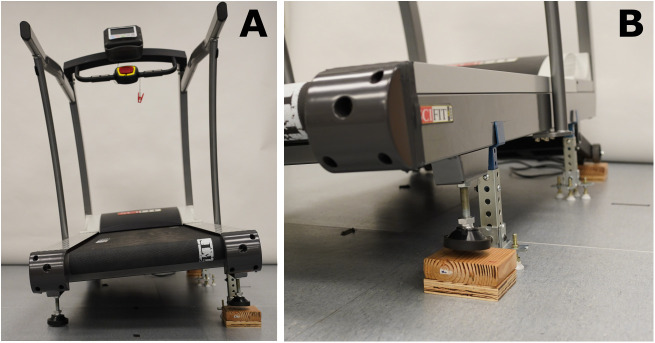
Cross-slope walking condition on Sci-Fit AC5000 treadmill. Cross-slope walking activities will be completed on this treadmill with one side set higher than the other.

**Fig 5:**
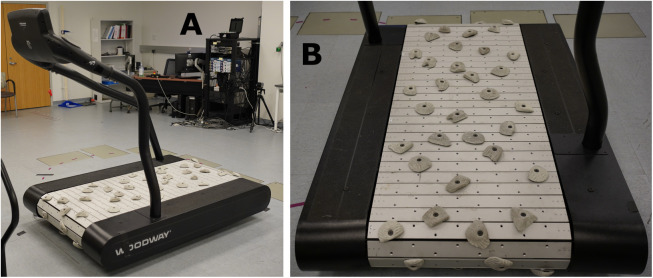
Customized Woodway 4Front Pro motorized treadmill to simulate uneven terrain. Walking activities at a comfortable walking speed will be completed on this treadmill.

During either visit 2 or 3 (participants are randomized to the order of *actual* and *emulated* trials – see below), participants will complete walking activities with three *actual* prosthetic feet in a randomized order. Participants will complete all the walking activities with one foot at a time prior to switching foot models. A prosthetist will optimize the alignment of each prosthetic foot (as would be done in the clinical setting) prior to walking activities. These walking activities will include walking on a Sci-Fit AC5000 treadmill at three different speeds, at an incline, and on a cross slope (Fig 4), and walking on uneven terrain on a customized Woodway 4Front Pro motorized treadmill with custom built slats with a series of 69 rock climbing holds which equates to 29.3 rocks/m^2^, placed in a quasi-random orientation. (Fig 5).

Participants can adjust their walking speeds as needed throughout the ‘test-drive’ visits, including selecting different comfortable walking speeds on the Sci-Fit and Woodway treadmills (all walking speeds will be finalized at the end of study visit 3 to ensure consistent within-participant walking speeds during biomechanical data collections). These in-laboratory activities are designed to simulate a range of environmental walking conditions often encountered in the community. Furthermore, we expect inclined walking to emphasize the mechanical behavior of the prosthetic feet in the sagittal plane, while cross-slopes walking will emphasize the coronal plane mechanical behavior of the prosthetic feet. We chose uneven ground walking to introduce terrain irregularities potentially affecting both the sagittal and coronal planes. Immediately following the walking activities, participants will rate their perceived stability, prosthetic foot preference, and perceived stiffness of each foot (Fig 1). Participants will rest as needed between each walking activity.

#### In-laboratory *emulated* prosthetic foot ‘test-drive’ trials.

Study methods will be the same as those described in the In-Laboratory *Actual* Prosthetic Foot ‘Test-Drive’ Trials, except for the following: 1) participants will walk with the PFE instead of the *actual* prosthetic feet and 2) participants will not walk on the uneven treadmill (because of geometric issues with the PFE foot pads interfacing with the rock features of the treadmill). Each participant will walk on three different *emulated* prosthetic feet (in a randomized order) corresponding to the three *actual* prosthetic feet.

#### Community trials.

At the end of visits 3, 4, and 5, a study prosthetist will fit participants with one of the three *actual* prosthetic feet in a randomized order. They will take each foot home and will be instructed to use it during regular daily activities for one week. After each week-long community trial, they will return to the laboratory for follow-up evaluation in the laboratory (visits 4, 5, and 6 respectively) at which time they will complete self-reported surveys, and performance-based measures (Fig 1). They will also complete the same walking activities as during the in-laboratory ‘test-drive’ trials while we collect biomechanical data via optical motion capture (detailed below). At the conclusion of the study (after completion of each participant’s sixth study visit activities), they will be re-fitted with their prescribed prosthetic foot.

#### Randomization and masking.

We will block randomize participants into different sequences of foot models that ensure each participant experiences a unique foot order across all study visits. Participants will also be randomized to either complete the *actual* ‘test-drive’ trials first (AE) or the *emulated* ‘test-drive’ trials first (EA). To mitigate participant expectation bias, we will mask participants to all foot conditions within each of the ‘test-drive’ trials and community trials. Feet will be referred to only as A, B, C, D, or E, and we will cover *actual* prosthetic feet with a sock to mask any identifiable markings. Data shared with our study biostatistician will be coded by foot condition (A, B, C).

### Outcomes

#### Self-reported measures.

Fig 1 details at which study visits we will collect each of the following self-reported measures.

Perceived mobility will be assessed using a custom version of the Prosthetic Limb Users Survey of Mobility (PLUS-M). This custom short form was designed to improve precision in a narrow range of measurement [28] and enhance our ability to detect differences between prosthetic feet. PLUS-M items are individually calibrated, therefore scores on the custom short form are directly comparable to other PLUS-M short forms and related normative scores. PLUS-M shows evidence of reliability across the range of measurement, excellent test-retest reliability [29], and convergent construct validity in LLP users [30]. Higher scores indicate a greater level of perceived mobility.

Balance confidence will be evaluated by administering a revised version of the Activities-specific Balance Confidence (ABC) scale [31,32]. We will use the 5-point ordinal scale and scoring methods recommended by Sakakibara et al. [31] (i.e., items scored 0 (*no confidence*) to 4 (*complete confidence*), with summary scores created from average item scores). The ABC scale has demonstrated evidence of good internal consistency, test-retest reliability, and convergent construct validity in LLP users [33]. Higher ABC scores are indicative of greater balance confidence.

Perceived satisfaction and limitations to participating in social roles and activities will be assessed by administering two Patient-Reported Outcomes Measurement Information System (PROMIS) surveys, the Satisfaction with Social Roles and Activities (SSRA) Short Form 4a, and the Ability to Participate in Social Roles and Activities (APSRA) Short Form 6a [34,35], respectively. The PROMIS-APSRA and PROMIS-SSRA surveys consist of six and four questions, respectively. Both are answered on a five-point scale, with higher scores indicating fewer perceived limitations and greater satisfaction participating in social roles, respectively.

Prosthetic foot satisfaction will be assessed using a revised version of the Trinity Amputation and Prosthesis Experience Scales Satisfaction (TAPES-Sat) subscale [36,37]. This survey consists of five items that are scored on a 1–3 scale, where higher scores indicate greater satisfaction. This measure has high internal consistency in LLP users [36].

During visit 1, we will document the number of falls and near-falls participants recalled in the past year, using questions from the LLP User Fall Event Survey [38]. A fall will be defined as “a loss of balance or sudden loss of support where your body lands on the ground, floor, or another object” [39]. A near-fall will be defined as “a loss of balance where you caught yourself or recovered your balance without landing on the ground, floor, or another object” [39]. We will also assess whether participants experience any falls or near-falls during each community trial (e.g., at visit 4, we will ask whether they experienced any falls or near-falls during the week they wore the first *actual* foot home; Fig 1). We will also ask participants about their fear of falling, using four single-item questions [40–43].

Immediately following each walking activity, participants will rate their perceived stability (both *actual* and *emulated*) on an eleven-point scale (0–10); at the end of all walking activities with a given foot, participants will rate their overall preference for each prosthetic foot. These rating scales (see S1 File) are based on the previously published prosthetic foot preference rating scales used for this purpose [44]. Higher scores indicate greater levels of perceived stability and preference, respectively. Participants will also rate their perception of the stiffness of each prosthetic foot on a five-point ordinal rating scale (scored from 1 (extremely soft) to 5 (extremely stiff); see S1 File) [44]. Finally participants will rank their relative preference of the feet after trialing all feet (i.e., after each set of ‘test-drive’ trials (*actual* and *emulated*) and at the end of all three community trials; Fig 1).

#### Performance-based measures.

Fig 1 details at which study visits we will collect each of the following performance-based measures.

We will evaluate balance using the Berg Balance Scale (BBS), which is a 14-item performance test that includes static and dynamic balance activities. We will score each from 0 to 4 as determined by the participant’s ability to complete the activity. We will generate a summary score from the sum of all BBS item scores; a higher score indicates better balance. The BBS has demonstrated high inter-rater reliability in LLP users [45] and strong convergent validity with the ABC scale [5].

We will assess walking endurance using the 2MWT, a brief submaximal measure of aerobic capacity and walking performance [26]. Participants will walk at their fastest speed for two minutes on a level indoor walkway. Participants may use an assistive device or walker, if needed. We will record the total distance walked during the 2MWT; a greater distance covered indicates greater walking endurance. The 2MWT has evidence of excellent test-retest and inter-rater reliability in LLP users and is recommended for both high- and low-mobility individuals [46,47]. As detailed above, we will sort participants into mobility groups based on their baseline 2MWT score.

We will evaluate multi-directional dynamic balance using the Four Square Step Test (FSST), which assesses an individual’s ability to step forward, sideways, and backwards over low objects [48]. The layout of the FSST is a cross of PVC pipes that forms four “squares” on the ground. To perform the FSST, participants must complete a clockwise and then a counterclockwise stepping sequence through four squares formed by the PVC pipes, with both feet stepping into each square. Participants will complete two timed trials after being offered an opportunity to complete a practice trial. We will use the faster of the two times for scoring; shorter FSST times are indicative of better balance performance and lower fall risk. The FSST has good discriminant validity and reliability among people with a LLP [49] and has also shown evidence of high inter-rater and test–retest reliability among people with a LLP [50–52].

We will use the Four Stage Balance Test (4SBT), which is comprised of four standing balance tasks that increase in difficulty, to assess static balance [53]. These tasks include 1) standing with feet side-by-side, 2) standing with one foot placed in the instep of the other, 3) standing with tandem stance (i.e., one foot directly in front of the other), and 4) standing on one foot. We will time each task for up to ten seconds. If a participant is unable to stand in any of these positions for ten seconds, the test ends immediately, and we add their times on each task for their final score. An inability to complete at least three of the 4SBT tasks (i.e., total time > 30s) has been shown to be associated with an increased risk of falling [53]. The 4SBT has been used in LLP users as part of previous studies [54,55].

We will evaluate lateral balance using the Narrowing Beam Walking Test (NBWT) [56]. The NBWT is a performance-based test developed to challenge lateral balance by constraining step width and/or reducing the support surface [57]. To perform the NBWT participants will cross their arms over their trunk and walk along a narrowing beam that consists of four fixed width beam segments, each 1.83 meters in length and 3.8 cm in height. The beam segments become increasingly narrow in width, from 18.6 cm to 4.0 cm [57]. Consistent with developer guidelines, participants will attempt five trials, with each trial ending when the participant either uncrosses their arms, steps off the beam, or walks its full length, whichever comes first [57]. We will score NBWT performance based on the normalized distance walked (i.e., the mean distance walked across each trial relative to the overall beam length) during trials 3–5 [57]. Larger normalized distances are indicative of better balance performance. In LLP users, the NBWT exhibits evidence of construct validity with FSST and TUG and a moderate correlation with the ABC [56]. The NBWT also demonstrates excellent content validity, known groups construct validity, and diagnostic accuracy.

We will also use the L-Test, which is a modified version of the TUG, designed to assess many of facets of mobility [58], including transfers, walking and turning in LLP users. The L-Test setup consists of a chair and two cones forming an “L” shape: one cone placed three meters in front of the chair and the second cone seven meters to the right of the first. To complete the L-Test participants will stand from sitting in the chair and walk around the first cone, the second cone, and then the first cone again in the “L” shape” before returning to sit in the chair. Participants will each complete the L-Test three times, and the average of the three times will be their final score; a faster time indicates greater mobility. The L-Test has shown strong correlation with the TUG and 2MWT, and moderate correlation with the ABC. The L-Test has also shown evidence of high intra-rater and interrater reliability [58].

#### Biomechanical measures.

Throughout the walking activities during visits 4, 5, and 6 (i.e., after each one-week community trial with an *actual* prosthetic foot), we will collect biomechanical data. These data include kinematic data collected via motion capture using retroreflective markers on body segments and joints. Using the positional and kinematic data collected via motion capture, we will calculate the mediolateral (ML) and anteroposterior (AP) margins of stability (MoS) during each step [59,60]. We will also measure spatiotemporal gait outcomes (e.g., step width and time asymmetry). When available, we will also collect kinetic data via an in-ground instrumented split-belt AMTI treadmill, which we can use to explore secondary outcomes of lower-limb and pelvis motions, moments, and powers to better understand compensatory strategies used during different walking conditions (Fig 1).

### Statistical analysis

To test the correlations between prosthetic foot stiffness and perceived and actual stability, we will perform linear mixed effects regression to assess the association between self-reported measures, performance-based measures, or biomechanical measures of stability (dependent variables) and prosthetic foot (independent fixed effect) with random effects for study participant. To assess whether short, in-laboratory prosthetic foot ‘test-drive’ trials are predictive of longer-term preference and stability while walking on *actual* prosthetic feet, linear mixed effects regression will test for correlation between self-reported measures while walking on the *actual* and *emulated* prosthetic feet (independent fixed variables) during ‘test-drive’ trials and the self-reported, performance-based, and biomechanical measures after the one-week community trials of the *actual* feet (dependent variables).

#### Power analysis.

We performed power analyses for two of the primary study variables—NBWT and MoS—using pilot data collected on unilateral, transtibial prosthesis users walking with a multiaxial/split-toe prosthetic foot (n = 34) and with a solid keel foot (n = 11). Participants using the former foot walked a mean 3.4 ft farther than participants using the solid keel foot, with between and within participant standard deviation (SD) 4.1 and 2.7 ft, respectively. Using these estimates, we carried out a power analysis on the NBWT scores, we simulated 5000 datasets of NBWT with sample size equal to 40 and 50 participants, each wearing all 3 feet (within-participant design), and with 1 mean pair-wise difference in NBWT of 2 ft; smaller differences than found in the pilot data as that study estimated between-participant differences which tend to be larger. For each dataset, linear mixed effects regression was carried out as described in the methods section, and power was estimated based on a type 1 error of .01, to account for multiple comparisons. We estimate 81% and 91% power to detect differences in NBWT of 2 ft for sample sizes of 40 and 50 respectively. Using estimates obtained from Gates et al (2013) [59], we simulated MoS in participants with transtibial amputation walking on uneven surfaces, using 8.3 cm, 1.9 cm and 1.0 cm as the mean, between-participant and within-participant SDs respectively using a similar procedure as above. We estimate 98% power to estimate a pair-wise difference between prosthetic feet of 1 cm for 40 participants, and 87% power to estimate a difference of 0.7 cm for 50 participants. Accounting for potential attrition, we will aim to recruit 60 participants (30 at each site), so that even if 20% of the recruited participants drop out, we will still have enough participants for adequate statistical power.

### Ethics approval

This study protocol was approved by the Veterans Affairs Central Institutional Review Board (#1618304). The original approval was granted on May 14, 2021, with the most recent amendment approval on September 10, 2024.

## Discussion

We aim to investigate how sagittal and coronal plane prosthetic foot stiffness affects the stability of LLP users while walking on different terrains. We also aim to investigate how well self-reported stability and foot preference during brief ‘test-drive’ trials of both *actual* and *emulated* prosthetic feet in the laboratory can predict longer-term self-reported, performance-based, and biomechanical measures with the same *actual* prosthetic feet after a one-week accommodation period in the community. We hypothesize that feet with the lower sagittal plane stiffness will correlate with greater perceived and measured stability while walking on inclines, while feet with lower coronal plane stiffness will correlate with greater perceived and measured stability while walking on cross-slopes and uneven ground. We also hypothesize that participants’ initial perceived stability with each *actual* and *emulated* foot condition during ‘test-drive’ trials will correlate with their balance confidence and stability after a one-week community trial.

The proposed study has key strengths, primarily in the steps we have taken to eliminate biases. Because many participants may have prior experience with one or more of these prosthetic feet, we will mask any identifiable markings on the feet to mitigate expectation bias on behalf of our participants. We will also randomize the order in which our participants will wear each of the feet to prevent a learning effect on their performance with each foot.

A potential limitation of the initial aim (correlating prosthetic foot stiffness with stability outcomes during walking activities) is that the proposed walking activities are mainly restricted to in-laboratory treadmill walking, which has been shown to elicit different walking patterns than unconstrained walking [61]. However, the walking activities in this study encompass a wide range of terrains and simulated environments, while the vast majority of previous studies on the effect of prosthetic foot properties are limited to just one or two walking activities. Further, treadmill walking will allow us to record longer bouts of steady state walking in our motion capture laboratory than would otherwise be possible during overground walking. Another potential limitation of this study is specific to the PFE in the second aim; it only emulates the forefoot stiffness of the *actual* prosthetic feet and the heel stiffness is replicated primarily via a passive heel spring. While the forefoot stiffness of a prosthetic foot is not the only aspect affecting individuals’ stability and preference, it has been shown to be a fundamentally important factor in both outcomes.

The results of this research have the potential to help guide clinical prosthetic foot selection as part of prosthesis prescription by providing evidence that matches mechanical properties of prosthetic feet (sagittal and coronal plane stiffness) with stability and mobility outcomes in LLP users. Furthermore, this research has the potential to guide a more patient-centered approach toward prosthesis prescription by enabling LLP users to ‘test-drive’ prosthetic feet to provide experiential input. The overall goal of this research is therefore to optimize stability and balance-related outcomes, to minimize falls, and to maximize satisfaction in LLP users.

## Supporting information

S1 File Surveys. This supplemental file includes the surveys that will be used to collect self-reported data of participants’ perceived stability, balance confidence, and prosthetic foot preference during participation in the study.
(PDF)

S2 File SPIRIT checklist. This supplemental file includes the completed checklist which is a guideline for protocols of clinical trials.
(DOCX)

S3 File Approved protocol. This supplemental file includes the IRB-approved protocol for this study.
(PDF)
