# Supplementary material for: Assessing the effects of prosthetic foot stiffness and foot preference on stability, balance confidence, and satisfaction in transtibial prosthesis users: Protocol for a randomized, participant-masked crossover trial using a ‘test-drive’ strategy
Source: PLoS One. 2025 Oct 22;20(10):e0334497. doi: 10.1371/journal.pone.0334497 (PMC12543115; doi:10.1371/journal.pone.0334497)
Supplement: S1 File — This supplemental file includes the surveys that will be used to collect self-reported data of participants’ perceived stability, balance confidence, and prosthetic foot preference during participation in the study. (PDF) [file pone.0334497.s001.pdf]

Prosthetic Foot Rating Scales

1. Rate your ability to walk at a comfortable speed when using this prosthetic foot:

☐	☐	☐	☐	☐	☐	☐	☐	☐	☐	☐
0	1	2	3	4	5	6	7	8	9	10
Unable to do										Without any difficulty

2. Rate your ability to walk at a slow speed when using this prosthetic foot:

☐	☐	☐	☐	☐	☐	☐	☐	☐	☐	☐
0	1	2	3	4	5	6	7	8	9	10
Unable to do										Without any difficulty

3. Rate your ability to walk at a fast speed when using this prosthetic foot:

☐	☐	☐	☐	☐	☐	☐	☐	☐	☐	☐
0	1	2	3	4	5	6	7	8	9	10
Unable to do										Without any difficulty

4. Rate your ability to walk uphill when using this prosthetic foot:

☐	☐	☐	☐	☐	☐	☐	☐	☐	☐	☐
0	1	2	3	4	5	6	7	8	9	10
Unable to do										Without any difficulty

5. Rate your ability to <u>stand</u> on the cross-slope (prosthetic foot <u>higher</u>) when using this prosthetic foot:

☐	☐	☐	☐	☐	☐	☐	☐	☐	☐	☐
0<br>Unable to do	1	2	3	4	5	6	7	8	9	10<br>Without any difficulty

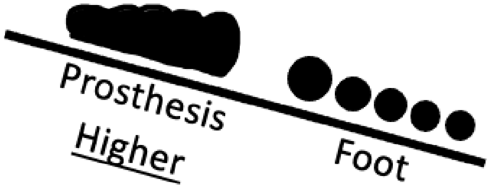

6. Rate your ability to <u>walk</u> on the cross-slope (prosthetic foot <u>higher</u>) when using this prosthetic foot:

☐	☐	☐	☐	☐	☐	☐	☐	☐	☐	☐
0<br>Unable to do	1	2	3	4	5	6	7	8	9	10<br>Without any difficulty

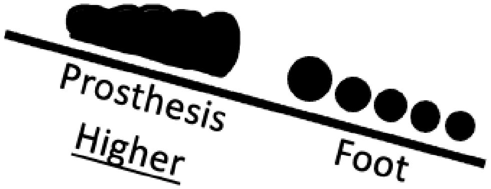

7. Rate your ability to <u>stand</u> on the cross-slope (prosthetic foot <u>lower</u>) when using this prosthetic foot:

☐	☐	☐	☐	☐	☐	☐	☐	☐	☐	☐
0	1	2	3	4	5	6	7	8	9	10
Unable to do										Without any difficulty

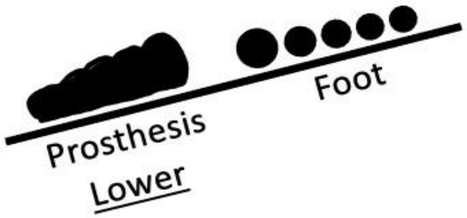

8. Rate your ability to <u>walk</u> on the cross-slope (prosthetic foot <u>lower</u>) when using this prosthetic foot:

☐	☐	☐	☐	☐	☐	☐	☐	☐	☐	☐
0	1	2	3	4	5	6	7	8	9	10
Unable to do										Without any difficulty

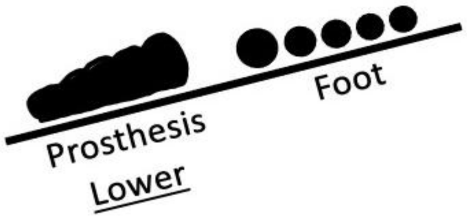

9. Rate your ability to walk on an uneven surface when using this prosthetic foot:

☐	☐	☐	☐	☐	☐	☐	☐	☐	☐	☐
0 Unable to do	1	2	3	4	5	6	7	8	9	10 Without any difficulty

10. Rate your overall satisfaction when using this prosthetic foot:

☐	☐	☐	☐	☐	☐	☐	☐	☐	☐	☐
0 Completely dissatisfied	1	2	3	4	5	6	7	8	9	10 Completely satisfied

11. If you had the option to use this prosthetic foot, rate how likely you'd be to choose it as a part of your daily prosthesis:

☐	☐	☐	☐	☐	☐	☐	☐	☐	☐	☐
0 Not at all likely	1	2	3	4	5	6	7	8	9	10 Extremely likely

12. How confident are you that you could keep your balance while walking with this prosthetic foot?

☐	☐	☐	☐	☐	☐	☐	☐	☐	☐	☐
0 Not at all confident	1	2	3	4	5	6	7	8	9	10 Extremely confident

13. Do you prefer this prosthetic foot or your own prescribed prosthetic foot?

☐	☐	☐	☐
This foot	My prescribed foot	No preference	Cannot Remember

Prosthetic Foot Perceived Stiffness Rating Scales

1. Rate how stiff you felt the toe/forefoot of this prosthetic foot was:

☐	☐	☐	☐	☐	☐
1	2	3	4	5	N/A
Extremely soft				Extremely stiff	Cannot Remember

2. Rate how stiff you felt the heel of this prosthetic foot was:

☐	☐	☐	☐	☐	☐
1	2	3	4	5	N/A
Extremely soft				Extremely stiff	Cannot Remember

3. Rate how stiff you felt this prosthetic foot was side-to-side:

☐	☐	☐	☐	☐	☐
1	2	3	4	5	N/A
Extremely soft				Extremely stiff	Cannot Remember

4. Rate how stiff you felt this prosthetic foot was overall:

☐	☐	☐	☐	☐	☐
1	2	3	4	5	N/A
Extremely soft				Extremely stiff	Cannot Remember
