# Supplementary material for: Assessing the effects of prosthetic foot stiffness and foot preference on stability, balance confidence, and satisfaction in transtibial prosthesis users: Protocol for a randomized, participant-masked crossover trial using a ‘test-drive’ strategy
Source: PLoS One. 2025 Oct 22;20(10):e0334497. doi: 10.1371/journal.pone.0334497 (PMC12543115; doi:10.1371/journal.pone.0334497)
Supplement: S3 File — This supplemental file includes the IRB-approved protocol for this study. (PDF) [file pone.0334497.s003.pdf]

A Prosthetic Foot Test-Drive Strategy for Improving Stability and Falls-Related Outcomes in Veterans With Leg Amputations

VAIRRS/IRBNet ID: 1618304

Funding Agency: Department of Defense

Principal Investigator/Study Chair: David Morgenroth, MD

Version 6; 08/30/2024

List of Abbreviations

2MWT – Two Minute Walk Test

4SBT – Four Stage Balance Test

ABC – Activity Balance Confidence

AE – adverse event

APSRA – PROMIS-APSRA, Patient-Reported Outcomes Measurement Information System Ability to Participate in Social Roles and Activities

BBS – Berg Balance Scale

CCTV – closed-circuit television

DoD – Department of Defense

FSST – The Four-Square Step Test

HM – high mobility

ISSO – information system security officer

LLA – lower limb amputation

LM – low mobility MVAHCS – Minneapolis Veterans Administration Health Care System

NBWT – Narrowing Beam Walking Test

NSR – non-significant risk

PFE – prosthetic foot emulator

PHI – protected health information

PLUS-M – Prosthetic Limb Users Survey of Mobility

RAC – Regional Amputation Center

SAE – serious adverse event

TAPES-R – Trinity Amputation and Prosthesis Experience (revised scales)

TTA – transtibial amputation (below-knee)

VAPSHCS – Veterans Administration Puget Sound Health Care System

Protocol Title: A Prosthetic Foot Test-Drive Strategy for Improving Stability and Falls-Related Outcomes in Veterans with Leg Amputations

1.0 Objectives

***Specific Aim 1*: Determine the effects of commercial prosthetic feet of varying stiffness on stability and falls-related outcomes in Veterans with TTA**

H1.1: Prosthetic feet with the lowest coronal plane stiffness will correlate with greater self-reported (i.e., Perceived Stability) and biomechanical (i.e., Medial/Lateral Margin of Stability (MOS)) stability outcomes during walking on uneven ground and cross-slopes, as well as greater balance confidence (i.e., ABC), performance-based stability (i.e., NBWT and Four Square Step Test (FSST)), and less activity restriction (i.e., PROMIS Ability to Participate in Social Roles and Activities (APSRA)) after community use.

H1.2: Prosthetic feet with the lowest sagittal plane stiffness will correlate with greater self-reported (i.e., Perceived Stability) and biomechanical (i.e., Anterior/Posterior MOS) stability outcomes during walking on inclines, as well as greater balance confidence (i.e., ABC) and stability (i.e., NBWT and FSST), and less activity restriction (i.e., PROMIS APSRA) after community use.

***Specific Aim 2*: Determine whether a multiaxial prosthetic foot emulator can be used to predict stability and balance-confidence outcomes with corresponding commercial prosthetic feet in Veterans with TTA.**

H2.1: Participants' initial perceived stability with each emulated foot will correlate with their perceived stability with the corresponding actual foot at follow-up.
H2.2: Participants' initial perceived stability with each emulated foot will correlate with their balance confidence (i.e., ABC) and stability (i.e., NBWT and FSST) in the corresponding actual foot at follow-up.

***Specific Aim 3*: Determine whether a brief trial of commercial prosthetic feet can predict longer-term stability and balance-confidence outcomes in Veterans with TTA.**

H3.1: Participants' initial perceived stability with each actual foot (relative to other feet) will correlate with their perceived stability with the corresponding foot at follow-up.

H3.2: Participants' initial perceived stability with each actual foot will correlate with their balance confidence (i.e., ABC) and stability (i.e., NBWT and FSST) in the corresponding foot at follow-up.

2.0 Study Procedures

2.1 Study Design

Study Population

Participants will be men and women, age 18 or over, with a unilateral TTA that have been fit with a prosthesis and use it on a regular basis (full inclusion/ exclusion criteria are listed below). Participants will be recruited at two sites: VA Puget Sound and Minneapolis VA. We will conduct a participant-blinded cross-over study with repeated measurements. Data collection methods will be identical at both sites. Participants will walk in the laboratory with the PFE and with corresponding/matched, commercial prosthetic feet under various walking conditions (e.g. level ground, incline, cross slopes, uneven ground). Participants will then wear each of the commercial feet at home and in the community for one-week periods. Participants will then return to the laboratory for follow-up visits after each one-week trial period to assess foot preference, stiffness perception, balance confidence, activity restrictions, stability, and falls-related outcomes in order to determine whether the PFE and/or a brief trial of commercial feet in the laboratory are able to predict mobility, stability, and falls-related outcomes after one week of use in the home and community setting.

No exclusions based on sex, race, or ethnic status will be applied.

**Study Groups and sites (enrollment)**	
Seattle VA: Participants with transtibial amputation	up to 50
Minneapolis VA: Participants with transtibial amputation	up to 50
**Total VA sites**	**up to 100**

Experimental Design

<u>Visits and length of participation</u>

Participants will be asked to participate in up to six study visits at the respective VA data collection site over approximately two to three months (See **Figure 1**). We expect each study visit to take approximately 3 hours, although there will

likely be some variability across participants. We will ensure that participants have adequate time to rest between walking sessions. If for any reason we find that data are missing or corrupted we may ask participants to return for another visit to recapture the data. We will make every effort to keep “on time” with the one-week follow up visit windows, however flexibility for participants’ schedules will be allowed and any variations in time will be noted as part of the data set.

Risks and Risk Management

Potential physical risks: All of the commercially available prosthetic feet used in this study are commonly prescribed. The prosthetic foot emulator also mimics the mechanical properties of these commonly prescribed feet. Participants will wear their prescribed prosthetic socket and suspension system throughout the entire study. Therefore, the physical risks associated with participation in this study only represent a minimal increase from those encountered in daily life of the participant population, and include:

- Trip and/or fall resulting from movement during study procedures and/or study provided prosthetic component failure or malfunction, leading to minor soft tissue injury (e.g., soreness, bruising, scrape) or other minor mechanical injury (e.g., knee or ankle joint soreness)

- Fatigue, minor muscle ache, or other soft tissue irritation from walking with an unfamiliar prosthetic foot or from wearing the tight-fitting body suit during visits 4-6.

Physical risk management: These risks will be minimized by providing frequent rest breaks during study sessions, providing prosthetic foot acclimation periods during study sessions, fitting participants with commonly-prescribed commercially available prosthetic feet that are specific to their mobility level (low or high), conducting walking tests at participants’ self-selected speeds, and having experienced certified prosthetists conduct all prosthetic fittings. Since participants will have a chance to wear each of the commercially available feet for at least an hour in the laboratory setting with an optimized alignment, we do not expect any problems to arise during the one week at home portions of the study. However, if a participant becomes uncomfortable with the study foot for any reason during the course of the study, they will be provided the opportunity to meet with our study prosthetist who will assess the study foot and alignment and make adjustments as necessary. If not feasible to visit the study prosthetist, the study prosthetist can recommend that the participant see their own, or a local prosthetist, for the alignment assessment and change. A note will be made by the study prosthetist to document this necessity. If a participant is not comfortable wearing any of the study prosthetic feet, they can opt out and we will replace the study foot with their originally prescribed prosthetic foot and study participation can be terminated as per participant request at any time.

To minimize the risks of falls or injury, the treadmills are equipped with safety handrails, and emergency stop capabilities. Participants will also be offered the option to be fit with additional supports (e.g., safety harness that is attached to the ceiling of the lab or a gait belt) while performing walking tests on the treadmill(s) while using the prosthetic foot emulator or the commercially available study feet. If a participant declines to be fitted with the extra safety equipment but appears unsteady or experiences a fall, we will request that they wear it if they wish to continue in the study. During the balance tests, two study personnel will also be present and stay close to the participant to ensure safety during these activities. Participants will also be able to skip any or all of the performance-based clinical tests should they not feel comfortable completing them. This will not disqualify them from participating in the rest of the study.

We do not anticipate safety problems associated with the PFE, however we will follow precautions to protect participants and have taken several design measures to prevent problems from arising:

(a) Standard Socket Emulator Interface: All loads delivered by the prosthesis emulator will be similar in magnitude and direction to those generated by off-the-shelf prosthetic devices. These loads will be transmitted through subjects' standard prescribed socket interface.

(b) Limited Load Direction: The platform cannot produce unusual loads on, e.g., ankle or knee joints, and cannot produce loads on the body when the foot is off of the ground, e.g. during swing.

(c) Mechanical Limits and Hardware Stops: To prevent emulator forces from becoming too large under any circumstances, we have implemented force-limiting points along the transmission from the motor to the end-effector. Before the emulator could apply forces large enough to cause injury, one of these force-limiting points would cause the device to become passive and the participant could stop walking without issue.

(d) Electrical Hardware Stop: All trials will have an emergency switch available that, when pushed, disconnects all power from the device. These switches allow participants or experimenters to stop the experiment in cases where co-robot behavior seems to present a potential risk, even though no negative events have occurred in prior studies with the emulator.

(e) Software Limits: Software limits have also been included, so as to prevent commanded torques and powers from becoming too large, and also to prevent the emulator from behaving in ways that might lead to mechanical failure.

(f) Extensive Pre-Trial Tests: To ensure proper mechanical function of the device and safety measures, we always perform pre-trial and benchtop testing prior to implementation of any modifications or upgrades to the emulator. When

changes are made to the emulator structure or controller, we test each safety feature and perform both passive (motors unpowered) and powered (motors on) software checks on all commanded torques and velocities.

Potential psychological risks: Please note, the psychological risks are minimal, the information collected is similar to that encountered in daily life or during the conduct of routine medical care.

- Participants may experience mild stress when acclimating to an unfamiliar prosthetic foot.
- Participants may find it inconvenient or frustrating to schedule and attend multiple study visits during their participation (6 total visits).
- Participants may find some the of interview questions uncomfortable or embarrassing and can opt not to respond to any question

Psychological risk management: Participants do not have to answer any questions that they do not want to and they may withdraw from the study at any time.

Privacy and Confidentiality

All engaged participating sites will safeguard data as required by VA information security policies.

See section 2.5 below for data collection procedures.

All participating sites will use the most current version of this study protocol. If these procedures are revised, the PI/SC will notify the site-specific Principal Investigator at each participating site and provide the revised version. All necessary local facility approvals will be obtained, if required, before the amendment or modification is implemented at each participating site.

2.2 Recruitment Methods

Please note that all references in this section to in-person contact/initial-screening will follow the Talking Points, all phone calls for contact/initial-screening will follow the Phone Script, and all references to approach letters and flyers refer to the Study Approach Letter and Flyers and will be tracked on the Recruitment Tracking spreadsheet.

Participants will be recruited at each of the two data collection sites: (1) VA Puget Sound Health Care System (VAPSHCS) and (2) Minneapolis VA Health Care System (MVAHCS). Potential participants may learn about the study from several sources: 1) targeted mailing or phone calls from medical record/database/registry, 2) study recruitment materials (e.g., flyers and brochures) posted throughout each data collection site and community-based outpatient prosthetic clinics (*copies of the letters of support will be submitted to*

the IRB as miscellaneous submissions, as they might be obtained), 3) clinician referral and study representative attending clinic, and 4) amputee-related newsletters.

We will recruit men and women, in the hope of maintaining at least a similar rate of representation found in our target population (i.e., Service members and Veterans with TTA). We anticipate that more men than women will be enrolled because there is a known sex difference, with more males than females (i.e., 65% male, 35% female) in populations of persons with major limb amputation, and this sex difference is substantially more extreme in service members and Veterans with LLA. Based on the results from previous research indicating challenges in recruiting and enrolling women with TTA, for this project we have explicitly included smaller prosthetic foot sizes and weight ranges that will hopefully improve our ability to include women participants who fulfill the recruitment criteria. Individuals will not be excluded on the basis of ethnicity or race. Individuals from all racial and ethnic groups will be recruited with the hope of maintaining a level of diversity that is representative of the surrounding community at each site.

<u>Medical Record/Database: Letter/Phone/In-person</u>

Designated research staff will screen relevant clinic lists (amputee rehabilitation, prosthetics, amputee support groups) in the VA electronic medical record system (CPRS or Cerner after planned VA transition to Cerner), and the Regional Amputation Center (RAC) database (a clinical database that includes a list of patients with an amputation who receive care) to identify potential participants. After review of relevant clinic lists in the VA electronic medical record system, designated staff will go to the clinic or contact providers on the phone to ask if the patient might be a good fit for the study. If the clinician agrees that the patient may be an appropriate study participant, during an appointment the clinician will ask the patient if she/he is interested in speaking with designated study staff; patients will be given a chance to opt out. For patients who are interested, designated study staff will speak to potential participants directly after a clinic visit and/or use the VA electronic medical record system to obtain potential participants' contact information (i.e., name, address, telephone number). For potential participants who learned about the study in person, designated study staff may make a follow-up approach phone call and/or send an approach letter to potential participants asking whether they are interested in participating in the study. If potential participants are unable to meet with designated study staff in-person then we will send an approach letter or call them to ask whether they are interested in learning additional information about the study. We may also search the VA electronic medical record system, and the RAC database, to identify individuals with a qualifying lower limb amputation and mail them the approach letter. If potential participants have not spoken with us within one week of the first call and/or mailing the approach letter, designated study staff will contact them by phone up to two more times (three times total) about this study. The approach letter will also include an "opt out" postcard. The opt-out postcard will have a

unique study recruitment identification code. The “opt-out” postcards and the exterior of the envelope for the approach letter do not include any information about the research project with any of the 18 HIPAA identifiers. If an individual returns the postcard to opt out they will not be approached about this study again.

Clinician Referral

Designated staff will inform providers working in relevant clinics (e.g., amputee rehabilitation, prosthetics, amputee support groups) about the study and inclusion/exclusion criteria so potential participants may contact the study team.

Designated staff may also inform providers working at local outpatient prosthetic clinics in the community about the study and inclusion/exclusion criteria so that potential participants may contact the study team (see list of clinics below, *copies of the letters of support will be submitted to the IRB as miscellaneous submissions, as they might be obtained*).

Flyers

Flyers will be posted in designated areas at each site, on the CCTV system and in publicly accessible locations in the community (e.g., public libraries, community centers, coffee shops). Flyers and study staff business cards will also be posted and distributed to potential participants at outpatient prosthetics clinics (listed below) in the community *(copies of the letters of support will be submitted to the IRB as miscellaneous submissions, as they might be obtained);* prosthetic office clinicians and support staff will direct interested individuals to contact us to learn more about the study.

DART/CDW

Another method of recruitment will be through IRB-approved screening of medical records via the Data Access Request Tracker (DART) request. To obtain the names and contact information from the VHA Corporate Data Warehouse (CDW), VAPSHCS or MVAHCS study staff may submit a data request via the DART workflow application. Using the contact information received from the DART request, study staff would make an approach phone call and/or send an approach letter to the potential participants asking whether they would like additional information about the study. If potential participants have not spoken with us within one week of the first call and/or of the mailing the approach letter, designated study staff will contact them by phone up to two more times.

Referral from Local Clinics

Center for Prosthetics and Orthotics (CPO), Inc., Cornerstone Prosthetics and Orthotics Inc., Hanger Clinic, Pacific Medical, and UW Medical Center Prosthetics near Seattle, WA and Tillges and Limb Lab near Minneapolis, MN may assist with

recruitment by offering recruitment information about the study to patients seen in their clinics who may be a good fit and are interested in learning more about it. They may post VA IRB approved flyers and distribute approved printed materials with information about the study and contact information for the VA Research Coordinator. Study staff contact information may also be provided to patients through their clinic staff. They will direct potential participants with questions about the research study to contact the VA Research Coordinator. We will keep the clinic updated on current inclusion and exclusion criteria.

<u>VA Puget Sound Center Registry: Letter/Phone</u>

At the VA Puget Sound, designated study staff may also identify potential participants using the VA Center for Limb Loss and MoBility Subject Registry VA IRB approved Registry (PI: Klute, #00433, David Morgenroth and Beth Halsne from the current project are study staff – see attachments 08a-c). The Registry contains contact information for participants who were screened for and/or participated in previous studies with our research group and agreed to be contacted for future studies. Designated study staff may make an approach phone call and/or send an approach letter to potential participants asking whether they are interested in the study. If potential participants have not spoken with us within one week of the first call and/or of the mailing the approach letter, designated study staff will contact them by phone up to two more times. The approach letter will also include an “opt out” postcard. The opt-out postcard will have a unique study recruitment identification code. The “opt-out” postcards and the exterior of the envelope for the approach letter do not include any information about the research project with any of the 18 HIPAA identifiers. If an individual returns the postcard to opt out they will not be approached about this study again. The link between the study recruitment identification code and their contact information will be kept in a separate password protected document on site at the VA Puget Sound (Seattle). Interested individuals will be screened for eligibility.

<u>Minneapolis VA: Letter/Phone</u>

Designated study staff may make an approach phone call and/or send an approach letter to potential participants asking whether they are interested in the study. If potential participants have not spoken with us within one week of the first call and/or of the mailing the approach letter, designated study staff will contact them by phone up to two more times. The approach letter will also include an “opt out” postcard. The opt-out postcard will have a unique study recruitment identification code. The “opt-out” postcards and the exterior of the envelope for the approach letter do not include any information about the research project with any of the 18 HIPAA identifiers. If an individual returns the postcard to opt out they will not be approached about this study again. The link between the study recruitment identification code and their contact information will be kept in a separate password protected document on site at the Minneapolis VA.

Recruitment Documentation

For all recruitment methods, each site will maintain a link between the study recruitment identification code and the contact information; these will be kept in a separate password protected documents at each site. These documents will also track the number of potential participants who were approached for recruitment, the source of recruitment, the dates and number of attempted phone calls, the results of screening for eligibility, and planned enrollment study visit date, as appropriate.

Participant Reimbursement

Participants will receive payment after each completed visit based on the schedule below. Payments will be dispersed by check by each site; checks will be mailed out as soon as possible after each visit, it may take up to 8 weeks for checks to be processed and mailed. The Seattle Institute for Biomedical and Clinical Research will process study payments for the Seattle VA. The Center for Veterans Research and Education will process study payments for the Minneapolis VA. Participants may choose to receive the payments in a pro-rated fashion following each study visit, or at the end of the study as a lump sum.

Visit 1 – In-person evaluation and assessment: $100
Visits 2-3 – Testing: $100 each
Visits 4-6 – Follow-up: $100 each

As noted above, individuals who are determined to be not eligible during the first visit will be compensated $50 for their effort.

If individuals have to travel more than 10 miles each direction to come for study visit(s), they will be eligible for reimbursement for miles traveled. This reimbursement will be calculated based on the IRS mileage rate ($0.56 for 2021) for the distance traveled to the study site (e.g., Seattle VA), with appropriate documentation, up to a maximum reimbursement for 100 miles traveled each direction. In addition, for the Seattle VA site only, travel within this radius requiring a ferry ride will be eligible for reimbursement. Eligible participants will be given the option to accept reimbursement for travel for each visit.

2.3 Informed Consent Procedures

A waiver of informed consent and HIPAA authorization will be used only for the recruitment and screening processes described above. A waiver of written documentation of informed consent and HIPAA authorization will be used in order to retain the preliminary eligibility screening information *(see eligibility criteria listed below)*. Informed consent will be obtained prior to enrollment in the study.

Informed Consent form

All participating sites will use the most current version of the combined informed consent and HIPAA authorization form. If the informed consent form is revised, the PI/SC will notify the PI's at each participating site and provide the revised draft of the consent form. All necessary approvals will be obtained before the amendment or modification is implemented at each participating site.

<u>Consent process</u>

Following initial screening, designated study staff and the participant may set up a phone call to review the informed consent process or a time to meet for the first visit to go through the informed consent process and obtain the necessary signatures. The combined informed consent and HIPAA authorization form may be mailed to individuals who pass initial screening in advance of the phone conversation or their first visit. Individuals who sign the combined informed consent and HIPAA authorization form in advance of their first in-person visit will bring the form with them to the first visit and study staff will make a copy for the participant. In the case that the individual forgets to bring the informed consent form with them, new copies will be provided and reviewed with the participant during the first visit. The informed consent process will take place in a private area, either on the phone or at the first visit, and in a discreet manner to protect the participant's privacy. Participants will be reminded that their participation is voluntary. Staff will go over the consent form with the participant and also give them time to read the form. Potential participants will also be asked if they need some time to consider their involvement before providing consent, and they will be given the opportunity to ask any questions related to the study before consenting. No attempt will be made to persuade individuals who decline participation. Designated study staff may not enroll participants if they arrive to the first visit and it becomes apparent that the participants may not be able to provide informed consent (e.g. subjects with impaired decision-making ability or the use of a legally authorized representative).

Designated staff at each site (as noted above) will conduct the informed consent process. We will avoid having the study PI/LSI conduct consent in order minimize the risk that individuals might feel obligated to participate. However, in the event the PI/LSI is the only team member available to conduct consent then she/he may do so. Finally, we will tell all potential participates that their decision about participation will not affect their clinical care in any way.

All study staff will complete the necessary human subjects protections training per VA policy. Additionally, the PI/SC and LSIs are responsible for training all applicable study staff at participating sites how to conduct the informed consent process. The PI/SC will also hold pre-study training conference calls, which will cover the informed consent process. LSIs are responsible for ensuring that their site study staff receive any additional training as necessary.

2.4 Inclusion/Exclusion Criteria

<u>Inclusion criteria</u>

- has a unilateral (one leg) transtibial (below-knee) amputation

- has used a prosthetic limb for walking for at least six months
- has a comfortably fitting prosthetic socket
- has a removable prosthetic foot attachment (i.e., is not rigidly attached to the back of the socket) so it can be used with study prosthetic feet
- be able to walk with a prosthetic limb sufficiently to participate in the experiment walking trials
- be age 18 years or older

<u>Exclusion criteria</u>
- contralateral limb or upper limb amputation that would interfere with completion of study activities
- are unable to use more than one of the test feet for any reason (e.g., excessively long residual limb that is not compatible with wearing study prosthetic feet)
- unable to walk under the minimal necessary study walking conditions in order to complete the study procedures without undo stress
- current surgical, neurological, rheumatologic, or lower limb musculoskeletal problem that significantly impairs ambulation (e.g., current ulcer, terminal illness)
- weight greater than 263lbs.
- inadequate cognitive or language function to consent to participate
- currently incarcerated
- impaired decision-making ability or the use of a legally authorized representative

2.5 Study Evaluations

<u>Data Collection</u>

There is an overview of the data collection presented in Figure 1. The methods are then described in detailed below. Study staff will be trained to carry out all of the protocol, including biomechanical gait data collection, and using the treadmills, the commercial prosthetic feet and PFE. This training may include onsite instruction and testing with equipment vendors and suppliers, as needed.

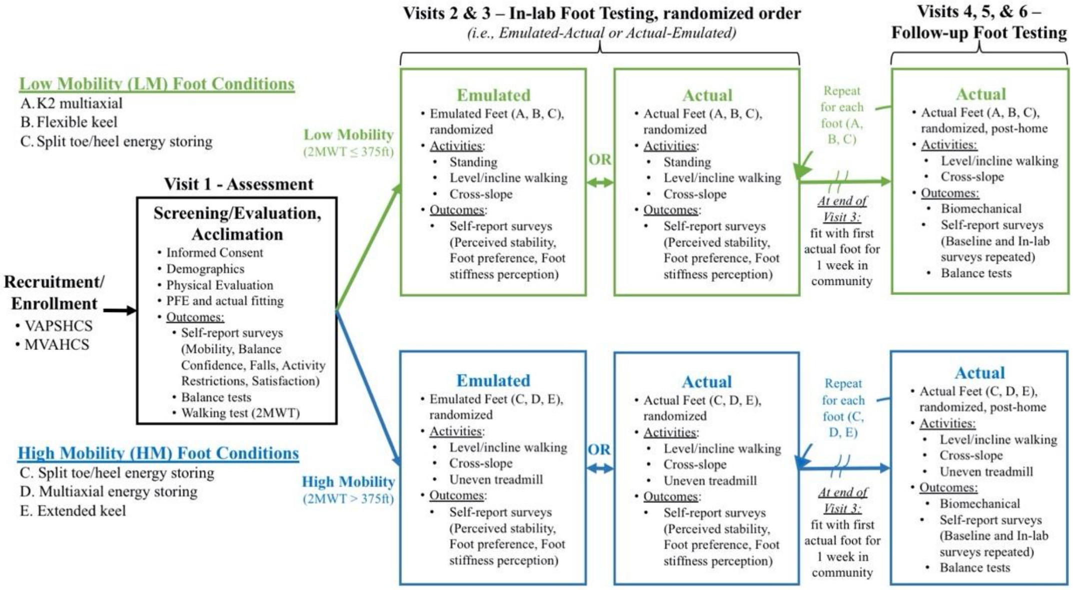

Figure 1. Schematic diagram of data collection study visits and outcome measures.

<u>Visit 1: Screening, Prosthetic Foot Emulator Fitting, and Assessment Visit (approximately 3 hours)</u>

After consent, participants will be assessed to determine if they can safely and successfully use the necessary study commercial prosthetic feet and the prosthetic foot emulator. For example, if a participant's residual limb length is too long to accommodate more than one of the prosthetic feet or if any other residual limb condition that precludes use of a prosthesis (e.g., ulcer) is present, she/he will be withdrawn from the study. Individuals who are determined to be not eligible will be compensated $50 for their effort.

Eligible participants will be asked to fill out the baseline self-report surveys; we will explain that they do not have to answer any questions they do not want to.

Participants will then be asked to complete a walking test called the Two-Minute Walk Test (2MWT) using their own prosthesis. They will be asked to walk continuously back and forth between two cones on level ground for 2 minutes; however, they may stop or take a seated rest break at any point during the test. Participants will be instructed to walk as quickly and safely as they can during this test. Study staff will walk alongside the participants to ensure safety during the test. Participants may use an assistive device, such as a cane or walker, when performing this test. We will record the total distance participants walked during the test.

In order to further assess the effect of the prosthetic foot type on balance and stability in people with LLA, we will conduct a set of baseline performance-based outcome measures (detailed below), in the following order, using their prescribed prosthesis. Participants will be encouraged to take as many rest breaks as needed throughout testing.

1. Berg Balance Scale (BBS): The BBS is a 14-task performance-based measure designed to assess static balance and fall risk in clinical populations. Each task is scored between 0-4, determined by ability to perform the assessed activity. These task scores are then summed to produce a final score. The BBS takes 10-20 minutes to complete. All tasks will be done while the participant is wearing their prescribed prosthetic foot.
2. The Four-Square Step Test (FSST): The FSST is a clinical outcome measure designed to assess dynamic balance. Participants are asked to step quickly in a 4-step pattern (i.e., forward, laterally, and backward) over canes (e.g., PVC piping) laid on the floor as per standard administration of the test. Participants complete the test twice while wearing their prescribed prosthetic foot, and the fastest of the two times required to complete the test is recorded. The FSST, including instructions, a practice trial, and the two timed trials, takes no more than 5 minutes to complete.
3. 4 Stage Balance Test (4SBT): The 4SBT is a performance-based test of static balance. There are up to four standing positions that get progressively more challenging to maintain for 10 seconds without moving their feet or needing support. The four positions are: feet side-by-side, placing instep of one shoe so it is touching the big toe of the other shoe; tandem stance of one shoe in front of the other with heel touching toe; and standing on one foot. Each position is described and demonstrated to the participant. Then, the participant may be guided and supported into assuming each correct position. When the participant is steady, they are timed to see how long they can maintain the position, but with close guarding ready to assist them if they should lose their balance and need to take a step. If the participant can hold a position for 10 seconds without moving their feet or needing support, they proceed to the next position. Participants are scored based on how many of the four stages they can safely complete.
4. Narrowing Beam Walking Test (NBWT): The NBWT is a performance-based test of balance for lower limb prosthesis users. It is comprised of four sets of low-to-the ground (i.e., approximately 4cm) beams that become increasingly narrow in width. Two practice trials and then three measured trials are performed with the distance walked on the beam averaged to produce a final score. The NBWT takes 3-5 minutes to complete. All trials will be done while the participant is wearing their prescribed prosthetic foot.
5. The L Test of Functional Mobility (L Test): The L test is a clinical measure designed to assess functional mobility. Participants will be asked to rise from a chair, walk 3 meters, turn at ninety degrees, walk 4 more meters and turn around, walk 4 meters, turn ninety degrees,

and return to the chair, and to a seated position. Time to complete the task is recorded. The L test takes 1-2 minutes to complete. One practice trial and three timed trials will be performed and the average time will be used for analysis. All trials will be done while the participant is wearing their prescribed prosthetic foot.

Participants will be asked to walk on the treadmill (e.g., AC5000M medical treadmill, SciFit, Tulsa, OK) with their prescribed prosthesis and to choose three walking speeds: comfortable, slow, and fast. Participants will choose these speeds by increasing and/or decreasing the speed on the treadmill. This will allow participants to practice walking on the treadmill before trying the study prosthetic feet. They will be allowed to hold onto the safety bars at any time on the treadmill if needed in order to keep their balance. They will also be offered the option of using additional supports (e.g., gait belt, overhead safety harness) while walking on the treadmill throughout the study.

Participants will be assigned to the low mobility group (LM) or the high mobility group (HM). LM vs. HM grouping will determine which 3 prosthetic foot conditions each participant will wear during the testing protocol. This grouping will be informed by the results of the participant’s 2MWT test, based on a cut-off distance of 113 meters as a guideline with clinical judgement. Although 113 meters translates to 371 feet, we use a cut-off of >375 feet for logistical purposes (i.e., distance between cones that participants will walk between is 75 feet). This is to ensure that participants will be using prosthetic feet that would likely be considered for their respective mobility levels in the clinical setting. For example, if a participant's 2MWT test score is marginal, the study research prosthetist will use other clinical indicators (e.g., balance, fatigue, quality of walking) to help inform the group assignment to ensure the participant can safely and comfortably complete the study activities for their respective group.

Please note: All prosthetic fittings for this study will be completed by a certified prosthetist, and they will be performed to meet normal clinical care standards. The treadmills described in the procedures below are equipped with safety rails and emergency stop features; participants will be given the option of using additional supports (e.g., gait belt, overhead safety harness) during the walking tests on this equipment.

The study research prosthetist will fit participants with a robotic ankle-foot prosthesis (the multiaxial prosthetic foot emulator, PFE). The PFE will be attached to participants’ current prosthetic socket in the same way that a regular prosthetic foot would be attached. The PFE can be programmed to operate in different modes that mimic the mechanical properties of different commercially available prosthetic feet. For this study, the PFE will be programmed with 5 foot modes – 3 high mobility feet and 3 low mobility feet (one foot mode is shared between high and low mobility groups). Designated research staff will use the computer software interface to switch the PFE between different foot modes and adjust hardware on the foot (e.g., adding small brass weights to mimic the weight of the corresponding prosthetic feet). Changing out the physical foot is not necessary. After participants are fitted with the PFE, we will give them time to

walk at their comfortable speed on the treadmill again while using the PFE in up to 3 different modes (3 high mobility vs. 3 low mobility modes depending on their 2MWT; see details above). Participants can choose to adjust their walking speed while walking with the PFE by increasing and/or decreasing the speed on the treadmill at any time. This will allow the participants to practice walking with the PFE and we will assess their walking ability to confirm whether the participant is a good fit for the study. Adjustments may be made to the PFE hardware or software settings in order to optimize the walking performance for each participant. If participants are not able to feel confident and stable on one or more of the modes, we will stop the session. We will also give participants time to rest at any point, and each time we switch foot modes. Participants may also be offered an opportunity to practice walking with the 3 different commercially available actual study feet.

At the end of the study visit, the prosthetist will refit the participants' prescribed prosthetic foot. The next study visit will be scheduled and a reminder slip can be given to the participant.

Visit 2: Randomization & First Initial Testing Visit – Emulator or Actual Feet (approximately 3 hours)

Based on the result of the 2MWT completed during Visit 1, participants have been assigned to the low mobility group (LM) or the high mobility group (HM) (see details above). At this visit, within each group (LM and HM) participants will be randomized to test either the PFE in the 3 foot modes for their group (LM vs. HM) or the 3 actual corresponding prosthetic feet for their group. Once randomized to PFE or actual feet, the order in which the different modes and actual feet will be tested will also be randomized; participants will be blinded to all PFE modes and actual foot conditions until the completion of the study. At visit 3 they will be tested under the other condition (e.g., if randomized to PFE testing during Visit 2, the participant would undergo testing with the corresponding actual feet during Visit 3).

The research prosthetist will fit participants with the assigned prosthetic feet (PFE or actual feet); participants will use their own prosthetic socket, interface and prosthetic suspension system. Participants will be asked to walk at their chosen comfortable speed on the treadmill while using the first randomly assigned PFE mode or actual foot until they feel comfortable. Adjustments may be made to the hardware or software settings in order to optimize the walking performance and comfort for each participant, if needed. If participants are not able to feel confident and stable on one or more of the PFE modes or on one of the actual feet, we will stop the session. We will also give participants time to rest at any point, and at every instance between switching modes or switching between actual feet. Participants will be able to take rest breaks at any time during the testing procedures and may choose to opt out of any of the tests at any time.

Please note: Participants will be allowed to hold onto the safety bars at any time on the treadmill(s) if needed in order to keep their balance. They will also be offered the option of using additional supports (e.g., gait belt, overhead safety harness) while walking on the treadmill(s) throughout the study.

When ready, all participants will be asked to walk on the treadmill for up to a minute at each of their previously-selected walking speeds: slow, comfortable, and fast. Participants will be asked to repeat these walking tasks with the second and third randomized PFE mode or actual foot, when ready. Study staff will set the PFE to the subsequent modes or the prosthetist will fit the subsequent feet.

Group 'HM' (high mobility): participants will then be asked to walk again for up to a minute at their selected comfortable walking speed, this time with the treadmill inclined up to 12% grade (~7º) (similar to going up a moderate to steep hill). This inclination is the maximum amount of the treadmill, and is less steep than what has been commonly used for people with lower limb amputations in previous studies. We will stop the amount of inclination before 12% grade or adjust their walking speed, if needed. Next they will be asked to stand then walk, at a comfortable walking speed, on cross slopes on each side of up to 8.5% grade (~5º) for up to a minute. This cross-slope inclination has been used in previous studies with this patient population. Finally, participants may be asked to walk at a comfortable walking speed on an uneven ground treadmill for up to one minute (Pro Slat Treadmill, Woodway, Waukesha, WI). For this treadmill, low-height rock holds resembling uneven ground have been fixed to the treadmill slats to present a challenging walking surface. This uneven treadmill surface is similar to conditions used previously to simulate walking in real-world uneven terrain conditions, including in people who have lower limb amputation. Participants will be offered a break prior to walking in each condition.

Group 'LM' (low mobility): participants will then be asked to walk again for up to a minute at their selected comfortable walking speed this time with the treadmill inclined up to 8.5% grade (~5º) (based on the Americans with Disabilities Act (ADA) compliant ramp inclination angle). This inclination has been commonly used for people with lower mobility and lower limb amputations in previous studies, based on ADA regulations. We will stop the amount of inclination before 8.5% grade or adjust their walking speed, if needed. Next, they will stand then walk on the treadmill with cross slopes on each side of up to 5% grade (~3º), and finally they will be offered the *option* of using the uneven ground treadmill for up to one minute.

When ready, participants will be asked to repeat these activities (e.g., inclined walking, cross slopes, uneven ground) with the second and third randomized PFE modes or actual feet. Between activities, participants will be given the opportunity to rest for as long as needed. During this time, they will be asked to complete a survey that includes questions about their preference, perceived stability, perceived foot stiffness, satisfaction and perceived mobility with the PFE modes or actual feet that they just walked with. These questions will occur following each activity as they complete it, or following all activities for items that refer to overall impressions about the foot that they just walked with.

Furthermore, during these visits biomechanical data may be collected using one of the following procedures: a 16-camera motion capture system (Vicon Motion Systems Ltd., Oxford, UK) to collect optical marker position data, or a set of inertial measurement units (IMUs; XSens, Movella Inc., Henderson, NV, USA) to collect acceleration data, which can be used to calculate positional data. If using the motion capture system, up to eighty reflective markers will be placed on anatomical locations (e.g., arms, legs, torso) and corresponding measurements will be taken (e.g., leg length, ankle width) according to the Vicon Full Body Plug-In Gait method. As is standard for gait biomechanical data collection, the infrared motion capture cameras will collect marker coordinate data during walking. If using the IMU system, up to twenty-five IMUs will be placed on anatomical locations (e.g., arms, legs, torso). These IMUs are commonly used, self-contained units that would be placed on participants in the same way as the reflective markers. No data will be shared with either Vicon Motion Systems Ltd. or Movella Inc. Embedded force platforms may also be used to collect synchronized ground reaction force data. The participants will be allowed to rest as much as needed between or during study activities. Dynamic stability with the foot conditions will be quantified during all walking activities from optical marker position data or IMU-based position data. The margins of stability (MOS), a commonly used biomechanical measure to assess dynamic stability in people with LLA, supposes that stable gait requires the center of mass (COM) to be controlled relative to the base of support (BOS), and the likelihood that the COM will successfully pass the leading foot can be assessed in the coronal and sagittal planes. Prosthetic foot stiffness properties in the sagittal and coronal planes may affect the COM velocity and BOS during prosthetic stance phase, especially in the presence of terrain challenges (e.g., cross-slopes and uneven treadmill), thus impacting the anterior-posterior and medial-lateral MOS. Minimum MOS in the coronal plane will be calculated based on the medial-lateral distance between the lateral border of the base of support (e.g., marker on 5th metatarsal head of the lead foot) and the vertical projection of the extrapolated COM. This technique is a modification of the original strategy proposed by Hof, et al. to define BOS based on optical markers or IMUs instead of center of pressure data, but it has a successful precedence in people with LLA under similar circumstances (e.g., variable terrains without force plates for center of pressure calculations). Similarly, anterior-posterior MOS will be calculated based on the vertical projection of the extrapolated COM relative to the posterior border of the BOS (e.g., heel marker), in accordance with published techniques in people with LLA. In calculating MOS, we will exclude strides in which participants hands are in contact with the handrail (as this would confound MOS calculations). Additionally, we will quantify handrail touches.

Once the data collection is completed, the prosthetist will refit participants' prescribed prosthetic foot. The next study visit will be scheduled and a reminder slip will be given to the participant.

Visit 3: Second Initial Testing Visit – Emulator or Actual Feet (approximately 3 hours)

At this visit participants will be asked to repeat the study procedures described above in Visit 2; they will complete the procedures using the equipment (PFE or actual feet) that they did not use during Visit 2 (based on randomization as described above).

At the end of this visit, the prosthetist will fit participants with one of the three actual feet that they used earlier; foot order will be randomized again and participants will remain blinded to foot condition (lightweight fabric will be secured around any potentially identifying features of the prosthetic foot using zip ties). Participants will wear the assigned study prosthetic foot (connected to their prescribed socket) for the next one week at home and in the community. Participants will be scheduled to return for a follow-up visit after this one-week period. Designated study staff will store the participants' prescribed prosthetic foot in a designated, secured cabinet within a locked office until they have completed the study or choose to stop their participation. Participants will be instructed to contact us if they have any concerns or discomfort associated with their prosthetic limb. If needed, participants can come in for prosthetic alignment adjustments during the 1 week in home/community test window. Alternatively, participants may also see their own prosthetist for adjustments, if needed, during the test window. In this case, study staff will make note of the necessity for the change and the clinic that the participant visited.

Note that prosthetic alignment will be optimized under all foot conditions.

Visits 4, 5 and 6: Follow-up Testing Visits (up to 4 hours each)

During each of these visits, participants will be asked to complete a survey that includes questions about their preference, stability, perceived foot stiffness, satisfaction, balance, activity restrictions, fear of falling, and mobility while using the study assigned prosthetic foot that they wore for the past week. During each of these visits, participants will be asked to repeat some of the performance-based balance-related outcome measures (i.e., FSST, 4SBT, NBWT, L test) (as described under Visit 1, with the only difference being that participants will be wearing the respective study prosthetic foot during visits 4, 5, and 6).

Furthermore, during these visits, a 16-camera motion capture system (Vicon Motion Systems Ltd., Oxford, UK) or an IMU system (XSens, Movella Inc., Henderson, NV, USA) may be used to collect positional data, using the same procedures described above in visits 2-3. Up to eighty reflective markers, or up to twenty-five IMUs, will be placed on anatomical locations (e.g., arms, legs, torso) and corresponding measurements will be taken (e.g., leg length, ankle width) according to the Vicon Full Body Plug-In Gait method. No data will be shared with Vicon Motion Systems Ltd. or Movella Inc. Participants may be asked to repeat some of the previous study procedures (i.e., walking conditions) while biomechanics data is being collected. Specifically, they may be asked to repeat

some of the previous study activities, including standing and walking trials overground and on the level, inclined, cross-slope, and uneven treadmill conditions (see details above, depending on their LM or HM group assignment) at their self-selected comfortable, slow, and fast walking speeds using the study foot that they have accommodated to following their one-week at home trial. As is standard for gait biomechanical data collection, the infrared motion capture cameras will collect marker coordinate data during walking if using the marker-based motion capture system. Embedded force platforms may also be used to collect synchronized ground reaction force data. The participants will be allowed to rest as much as needed between or during study activities.

At the end of Visits 4 and 5, the prosthetist will fit participants with one of the other remaining prosthetic feet (based on their randomized foot order and participants will remain blinded to foot condition). Participants will wear the assigned prosthetic foot (connected to their prescribed socket) for the next one week at home and in the community. Again, participants will be scheduled to return for a follow-up visit after one week. Participants will be reminded to contact us if they have any concerns or discomfort associated with their prosthetic limb. If needed, participants can come in for prosthetic alignment adjustments during the one-week in home/community test window. Should it not be feasible for the participant to come in to a study site (e.g., VAPSHCS) for alignment adjustments, study staff can recommend that the participant see their local prosthetist for any necessary changes. In this case, study staff will make note of the necessity for the change and the clinic that the participant visited.

Testing procedures will be repeated again at Visit 6. At the end of Visit 6, the prosthetist will re-fit the participants' prescribed prosthetic foot, which will conclude the testing sessions.

<u>Visit reminders</u>

Designated study staff may call and/or send a visit reminder email to participants prior to each study visit.

<u>VA Puget Sound only: Repository (optional)</u>

We may ask participants if they want to add their data (without identifiers) from this study to our data repository (Klute, MIRB #00493). Interested participants will be asked to sign a separate Consent Form for the repository.

<u>Photos and video recording</u>

With the participant's explicit consent, we may take video and photos during portions of this study, for documentation and use in dissemination efforts or educational materials. All videos and photos will exclude the participant's face and any identifying marks will be covered. If any identifiable features or marks are mistakenly captured they will be anonymized during data processing; the participant's face or other unique marks would be blurred prior to any use outside of the research team that captured the images. Videos will be recorded without

sound so that we do not capture any voice prints. If a participant's voice is accidently recorded that section of video would be altered prior to any use outside of the VA study team. Participants will be able to opt out of this activity and still participate in the study.

2.6 Data Analysis

Statistical Analysis

For both H1.1 and H1.2 (see Objectives section, 1.0 above for hypotheses details), linear mixed effects regression will be used to assess the association between Perceived Stability score, MOS, ABC, NBWT, or FSST (the dependent variables) and prosthetic foot (the independent fixed effect) with random effects for study participant. For H2.1 linear mixed effects regression will be used to test for correlation between Perceived Stability score for the emulated foot (the independent fixed effect) and follow-up Perceived Stability score for the corresponding actual foot (the dependent variable) with study participant as a random effect. For H2.2, similar models will be used to test for associations between the Perceived Stability score in the emulated foot (the independent fixed effect) and follow-up ABC, NBWT or FSST in the actual foot (the dependent variable). For H3.1 and H3.2, linear mixed effects regression will be carried out as above with initial Perceived Stability score as the independent fixed effect and Perceived Stability score, ABC, NBWT or FSST as the dependent variables with participant as the random effect. Linear mixed effects regression provides a more flexible approach to analyze repeated measures data compared to the traditional repeat measures models, with fewer model assumptions and the ability to incorporate data from participants who do not complete the study. These models account for non-independent data due to within-participant repeated measures by estimating separate between- and within-participant errors. For all models, mean and standard errors of the outcomes for each prosthetic foot and pair-wise mean differences and 95% confidence intervals (CI) across the three feet will be estimated and tested for significance.

Power Analysis: Sample size for this study was based on pilot data collected in support of Specific Aim 1. Power analysis was carried out for two of the primary study variables: NBWT and MOS (H1.1 and H1.2). For NBWT we obtained overall mean, between and within participant variances based on pilot data for 45 participants testing for differences in NBWT using a multiaxial/split-toe foot (n=34) vs. a solid keel foot (n=11). Participants using the former foot walked a mean 3.4 ft farther than participants using the solid keel foot, with between and within participant standard deviation (SD) 4.1 and 2.7, respectively. Using these estimates, we simulated 5000 datasets of NBWT with sample size equal to 40 and 50 participants, each wearing all 3 feet (within-participant design), and with 1 mean pair-wise difference in NBWT of 2 ft; smaller differences than found in the pilot data as that study estimated between-participant differences which tend to be larger. For each dataset, linear mixed effects regression was carried out as described in the methods section, and power was estimated based on a type 1

error of .01, as we have 5 outcomes of interest. We estimate 81% and 91% power to detect differences in NBWT of 2 ft. for sample sizes of 40 and 50 respectively. Using estimates obtained from Gates et al (2013),143 we simulated MOS in participants with TTA walking on uneven surfaces, using 8.3 cm, 1.9 cm and 1.0 cm as the mean, between-participant and within-participant SDs respectively using a similar procedure as above. We estimate 98% power to estimate a pair-wise difference between prosthetic feet of 1 cm for 40 participants, and 87% power to estimate a difference of 0.7 cm for 50 participants. Considering the potential for attrition, we will recruit 60 participants (30 at each site), so that even if 20% of the recruited participants drop out (a conservative estimate based on the <5% drop out rate in our current emulator study with the same number of study sessions), we will still have enough participants for adequate statistical power. This will also allow us to accommodate a higher drop-out rate and those who are deemed ineligible after signing consent during physical examination screening without a modification.

All statistical analysis will be performed by study staff with de-identified data. No PHI or VA sensitive data will be analyzed or shared across sites. De-identified data files will also be sent off-site to our biostatistician (VA research staff) and among staff and study collaborators across study sites. These will be sent via email and will only contain de-identified data. For example, de-identified data which has been exported from VA REDcap behind the VA firewall for data analysis. Only de-identified data will be disclosed outside of the VA. A copy of the de-identified data set will be sent off-site to our biostatistician, so that they may assist with data analyses. Our biostatistician will not have access to the key to the study codes/crosswalk or to any PHI. Our offsite collaborators (Drs. Andrew Sawers, Josh Caputo, and Carl Curran) may receive copies of data analyses summary reports (all de-identified) so that they may assist with data interpretation and manuscript preparation.

2.7 Withdrawal of Subjects

This is not a medication or treatment study therefore a strict process is not necessary because withdrawing or being terminated from this study will not have an impact on participant safety. A study clinician or the PI may withdraw a participant without their consent if he or she feels that it is not in a participant's best interest to continue in the study (e.g., safety concerns) or if they are unable to complete the study procedures. All data previously collected from participants who withdraw or are withdrawn will be kept and may be used in the study data analysis. Participants may also opt to withdraw at any time by informing the Research Coordinator and/or the PI at their site.

There is a possibility that some participants may decide to stop their participation, or be lost to follow up, while in possession of one of the commercial feet belonging to the study. If this circumstance arises, we will make a concerted effort to recover the prosthetic foot from the participant. Since the study feet are commercially available and prosthetic foot alignment will be optimized, there is

no concern that a retained study foot would cause a safety risk beyond standard prosthetic care. The participant’s prescribed prosthetic foot will be stored by the researchers at each site while the participant is wearing the study provided foot in the community.

3.0 Reporting

All safety information on Adverse Events (AE’s), Serious Adverse Events (SAEs), unanticipated events or problems involving risks to subjects, and protocol deviations will be collected. This information will be collected at study visits and whenever participants call to report a problem. This data will be collected for each participant throughout their invovlement in the study. All reporting requirements as noted in VHA Handbook 1508.01 will be followed.

The Local Site PI at each site will conduct timely reviews of all AEs (including anticipated AEs, SAEs, and Unanticipated AEs, U-SAEs, and other problems) and protocol deviations that occur at their site. These will be reported to the PI/SC and designated study staff as they occur/when the reporting site becomes aware of the events; this will allow the PI/SC and the medical monitor to track issues from all sites in a timely manner and take corrective action as needed. In addition, SAEs that have the potential to affect the overall study will be communicated to all engaged participating sites by the PI/SC. The Local Site PIs will report to the VA Central IRB and the PI/SC (Dr. Morgenroth) all problems involving previously unknown risks to participants or others and all local AEs and SAEs related to the research. Anticipated AEs related to the research will be reported to CIRB annually with the continuing review; if anticipated AEs occur at a higher than expected rate, the PI/SC and other investigators will reevaluate the study procedures and modifications will be prepared as needed. Unanticipated SAEs, protocol deviations and/or serious problems that are related to the research will be reported to the Central IRB within 5 business days of becoming aware of the event, per VA reporting requirements. Additionally, the local site PIs will also report to the VA Central IRB and PI/SC any issues of serious or continuing non-compliance within 5 days of becoming aware of the issue. If study data is improperly used or disclosed we will notify the ISSO and Privacy Officer immediately of becoming aware of the issue. This study does not have a Data Monitoring Committee.

Lastly, the PI/SC will ensure that the main study site (VA Puget Sound) keeps sites informed of any items that might be relevant to participant protection through regular teleconference meetings and/or email communications. The study coordinators and site PIs will meet (typically by telephone since site PIs are geographically disparate) at least once per month while data collection is ongoing).

The occurrence of 5 or more study-related AEs per site, or 2 or more study-related SAEs across all sites will trigger a review to compare frequency of AE’s and/or SAEs to rates reported in the literature. In the case of an SAE, an immediate review of the protocols by the LSI may be warranted at the time of occurrence. Although it is unlikely, if the review determines that the rate of AEs and SAEs is more prevalent in the study

than reported in the typical clinical setting in this patient population (per literature review) the research would be immediately suspended.

If we become aware of relevant findings or information that may affect participants' health or welfare we will contact them by phone and/or a letter to provide the information. The study does not have a data monitoring committee.
